# Association between gut microbiota and Hirschsprung disease: a bidirectional two-sample Mendelian randomization study

**DOI:** 10.3389/fmicb.2024.1366181

**Published:** 2024-03-07

**Authors:** Wei Liu, Hanlei Yan, Wanying Jia, Jingjing Huang, Zihao Fu, Wenyao Xu, Hui Yu, Weili Yang, Weikang Pan, Baijun Zheng, Yong Liu, Xinlin Chen, Ya Gao, Donghao Tian

**Affiliations:** ^1^Department of Pediatric Surgery, The Second Affiliated Hospital, Xi'an Jiaotong University, Xi'an, China; ^2^Institute of Neurobiology, Environment and Genes Related to Diseases Key Laboratory of Chinese Ministry of Education, Xi'an Jiaotong University, Xi'an, China

**Keywords:** Mendelian randomization analysis, Hirschsprung disease, gut microbiota, causality, bidirectional

## Abstract

**Background:**

Several studies have pointed to the critical role of gut microbiota (GM) and their metabolites in Hirschsprung disease (HSCR) pathogenesis. However, the detailed causal relationship between GM and HSCR remains unknown.

**Methods:**

In this study, we used two-sample Mendelian randomization (MR) analysis to investigate the causal relationship between GM and HSCR, based on the MiBioGen Consortium’s genome-wide association study (GWAS) and the GWAS Catalog’s HSCR data. Reverse MR analysis was performed subsequently, and the sensitivity analysis, Cochran’s Q-test, MR pleiotropy residual sum, outlier (MR-PRESSO), and the MR-Egger intercept were used to analyze heterogeneity or horizontal pleiotropy. 16S rDNA sequencing and targeted mass spectrometry were developed for initial validation.

**Results:**

In the forward MR analysis, inverse-variance weighted (IVW) estimates suggested that Eggerthella (OR: 2.66, 95%CI: 1.23–5.74, *p* = 0.01) was a risk factor for HSCR, while Peptococcus (OR: 0.37, 95%CI: 0.18–0.73, *p* = 0.004), Ruminococcus2 (OR: 0.32, 95%CI: 0.11–0.91, *p* = 0.03), Clostridiaceae1 (OR: 0.22, 95%CI: 0.06–0.78, *p* = 0.02), Mollicutes RF9 (OR: 0.27, 95%CI: 0.09–0.8, *p* = 0.02), Ruminococcaceae (OR: 0.16, 95%CI: 0.04–0.66, *p* = 0.01), and Paraprevotella (OR: 0.45, 95%CI: 0.21–0.98, *p* = 0.04) were protective factors for HSCR, which had no heterogeneity or horizontal pleiotropy. However, reverse MR analysis showed that HSCR (OR: 1.02, 95%CI: 1–1.03, *p* = 0.049) is the risk factor for Eggerthella. Furthermore, some of the above microbiota and short-chain fatty acids (SCFAs) were altered in HSCR, showing a correlation.

**Conclusion:**

Our analysis established the relationship between specific GM and HSCR, identifying specific bacteria as protective or risk factors. Significant microbiota and SCFAs were altered in HSCR, underlining the importance of further study and providing new insights into the pathogenesis and treatment.

## 1 Introduction

Hirschsprung disease (HSCR) is one of the most common forms of neurocristopathy in children, an intestinal peristaltic disorder showing obstruction and proximal megacolon that affects 1 in 5,000 human births. The pathogenesis is generally characterized by aganglionosis in the distal intestine (Gui et al., 2017; Fu et al., 2020). Most patients with heritable variation are diagnosed by rectal biopsy in the neonatal period due to significant constipation; however, a subset of children (~20%) develop symptoms until early childhood or even adolescence (De Lorijn et al., 2005). Surgery is the primary treatment method, but commonly gives rise to medical complications, such as enterocolitis (~35% after surgery), soiling, anastomotic stricture, or leak with abscess and chronic constipation, threatening crucially the growth and development of children (Heuckeroth, 2018).

It is well known that HSCR primally arises from genetic factors, such as RET, EDNRB, RARB, GATA2, and SOX10 mutations. Recently, increasing reports indicate that abnormal intestinal microenvironment contributed to the HSCR pathogenesis, especially gut microbiota (GM; Obata and Pachnis, 2016; Kuil et al., 2021; Vincent et al., 2023). The GM begins to colonize after birth, and changes significantly from the neonatal period to early childhood, which coincides with the development of the enteric nervous system (ENS; Backhed et al., 2015; Gritz and Bhandari, 2015; Yassour et al., 2016; Stewart et al., 2018). Some researchers transplanted fecal bacteria into the nerve-free colon and found that the GM played a positive role in ENS development (Obata and Pachnis, 2016). Meanwhile, 16S rDNA sequencing and targeted mass spectrometry were used to develop a detailed profile of how the gut microbiome and its metabolites benefited the aganglionosis animal model (Soret et al., 2020; Tian et al., 2023). However, it remains to be investigated to determine whether and which GM has a causal relationship with HSCR.

Mendelian randomization (MR) is a new statistical method that can explore the causal relationship between exposed factors and disease. MR follows Mendelian’s second law and relies on independent random assignment of genetic variants during meiosis to achieve randomization effects similar to randomized controlled trials (RCTs), which can effectively overcome the confounding factors (Ma et al., 2023; Vincent et al., 2023; Yan et al., 2023; Zanoaga et al., 2023). MR has been widely used to analyze the causal relationship between GM and spinal arthropathy (Lai et al., 2023), ophthalmic disease (Li and Lu, 2023), tumors (Jiang et al., 2023), psychiatric disorders (Zeng et al., 2023), and circulatory system disease (Dai et al., 2023). However, the relationship between GM and HSCR has rarely been studied based on MR methods.

In this study, a two-sample MR analysis was performed using pooled statistics from the genome-wide association study (GWAS) of MiBioGen and the GWAS Catalog to assess the causal relationship between GM and HSCR, which could provide new insights into the potential pathogenesis of how GM contributes to HSCR and the assignment of effective treatment strategies.

## 2 Methods

### 2.1 Overall study design

**Figure 1:**
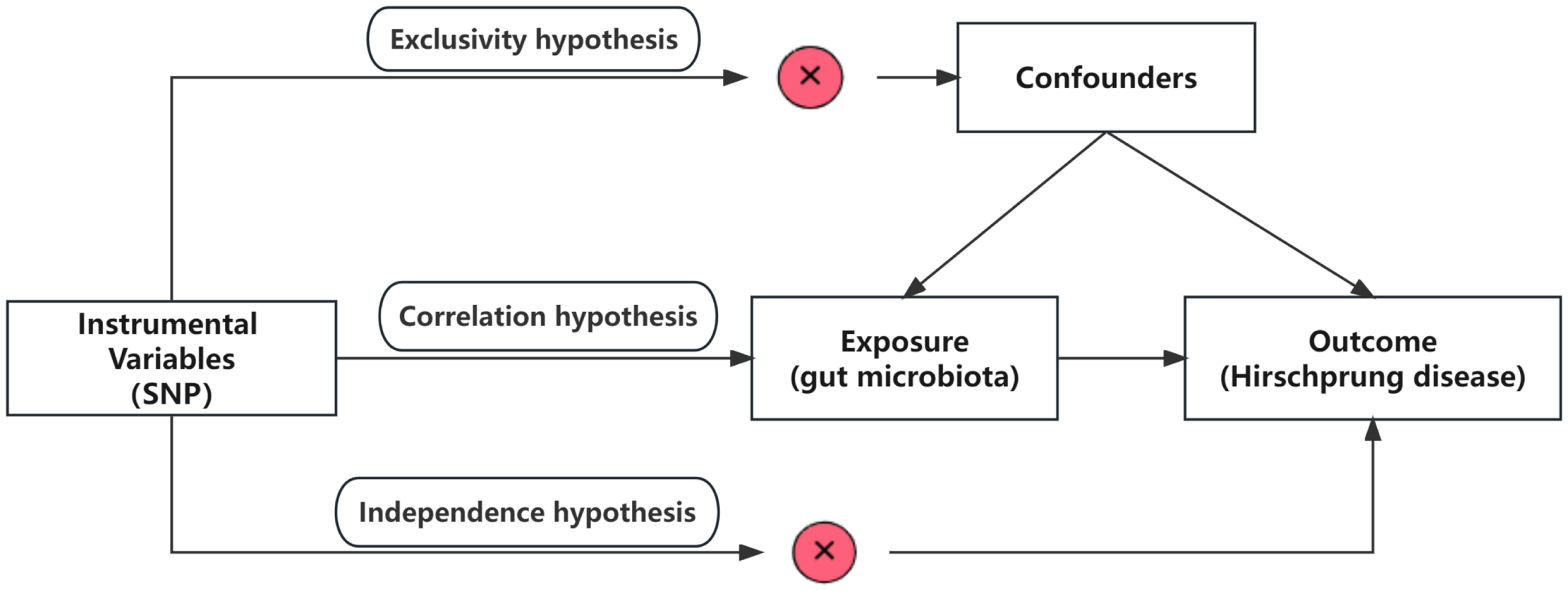
Overview of the study design. SNPs, single-nucleotide polymorphisms.

This study utilized pooled-level genetic data to conduct a bidirectional, two-sample MR analysis, aiming to test for the association between GM and HSCR. In order to reduce bias and obtain reliable results, we tried to satisfy the following three hypotheses when using MR analysis: (1) Correlation hypothesis: instrumental variables (IVs) are closely related to exposure; (2) Exclusivity hypothesis: IVs do not affect outcomes by other means; and (3) Independence hypothesis: IVs are not affected by confounding factors (Davies et al., 2018; Figure 1).

### 2.2 Data sources

Genetic variation for GM comes from the largest genome-wide meta-analysis of GM composition published by Kurilshikov et al. (2021) and MiBioGen Consortium (n.d.). They collected genome-wide genotypes and 16S fecal microbiome data from 18,340 individuals across 24 cohorts based on the MiBioGen consortium. A total of 196 taxa have been included in the study, classified under 5 biological categories: phylum, class, order, family, and genus. It should be noted that participants of European origin were exclusively considered, and 15 GM taxa (unknown family or genus) without specific species names were excluded from the analysis. GWAS summary statistics for HSCR were downloaded from the NHGRI-EBI GWAS Catalog (Sollis et al., 2023) on 10 November 2023 for study GCST005289 (Fadista et al., 2018). This study used the phenotype of HSCR and combined the most extensive collection of sporadic Hirschsprung cases to date, including 586 cases and 5,620 controls of European ancestry, which combined data from Denmark, the U.S., Finland, and Sweden. In the process of data analysis, strict quality control was carried out on the samples (Fadista et al., 2018).

### 2.3 Selection of instrumental variables in forward MR analysis

The following selection criteria were used to choose the IVs: (1) single-nucleotide polymorphisms (SNPs) associated with each genus at the locus-wide significance threshold (*p* < 1.0 × 10^−5^) were selected as potential IVs (Li et al., 2023); (2) 1000 Genomes project European samples data were used as the reference panel to calculate the linkage disequilibrium (LD) between the SNPs, and among those SNPs that had R^2^ < 0.001 (clumping window size = 10,000 kb), only the SNPs with the lowest *p*-values were retained (Fan et al., 2023); (3) SNPs with minor allele frequency (MAF) ≤ 0.01 were removed; and (4) when palindromic SNPs existed, the forward strand alleles were inferred using allele frequency information. (5) In order to control bias caused by IVs, the strength of each IV was evaluated using the formula F = β^2^/SE^2^ to calculate the F-statistic, where β represents the effect size of SNP on exposure, and SE represents the standard deviation of SNP on exposure (Burgess et al., 2011). If the corresponding F-statistic was >10, it was considered that there was no significant weak instrumental bias (Burgess et al., 2011). Power calculations were conducted with the online Power Calculator Tool (Burgess, 2014; Online sample size and power calculator for Mendelian randomization with a binary outcome, n.d.).

### 2.4 Mendelian randomization analysis

**Figure 2:**
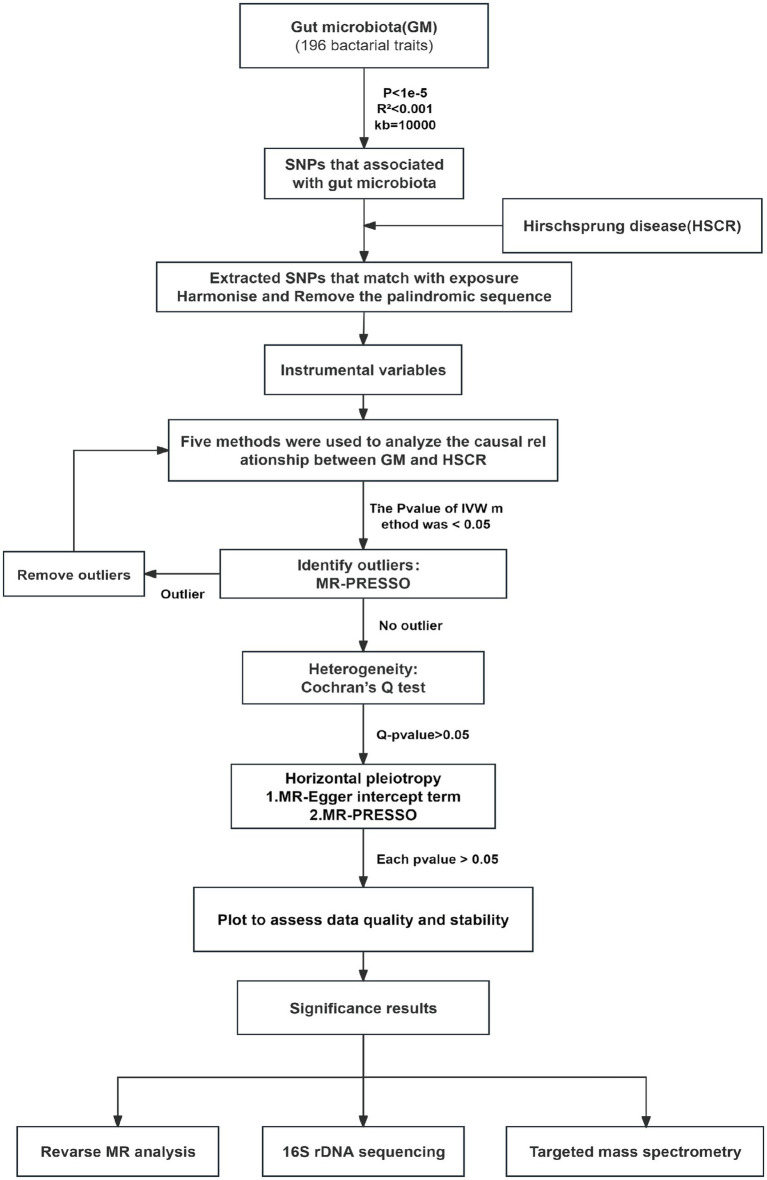
Flow chart of forward MR analysis. MR, Mendelian randomization; IVs, instrumental variable; IVW, inverse-variance weighted; SNPs, single-nucleotide polymorphisms.

MR is a statistical analysis method used for causal inference. It uses genetic variation as an IV to deduce the causal relationship between exposure and outcome, which can effectively avoid the influence of confounding bias in traditional epidemiological studies (Emdin et al., 2017). In this study, we analyzed the potential causal relationship between GM and HSCR with two-sample MR and strictly reduced the effect of confounders. The MR analysis process is shown in Figure 2. In the process of analyzing causal correlation, we used a variety of methods to evaluate it, including inverse-variance weighted (IVW; Burgess et al., 2017), MR-Egger test (Bowden et al., 2015), weighted median (WM), weighted mode (WMO), simple mode, and MR-PRESSO. The IVW method uses a meta-analysis method to combine Wald estimates of each SNP to summarize GM’s overall estimate of HSCR. If there is no horizontal pleiotropy, the IVW results are highly effective. MR-Egger tests the existence of the intercept term and uses it to evaluate pleiotropy. If the intercept term is very close to zero, then the MR-Egger regression model is very close to IVW, but if the intercept term is very different from zero, it indicates that there may be horizontal pleiotropy between these IVs. The WM is a method of considering the weight of the data when calculating the median, which can be used when the weight of the data should be considered more. The WMO takes into account the weights of the data points and is able to give a suitable mode if there are duplicate values with different weights. The simple mode can be used when each data point in the data set is weighted equally. MR-PRESSO improves the accuracy of causal associations by detecting and removing outliers. In the present study, we used the IVW method as the primary analysis method, while other methods were used to evaluate the stability of our results.

Heterogeneity was examined using Cochran’s Q-test, and a *p*-value of <0.05 was considered heterogeneous. The horizontal pleiotropy of IVs was measured by the MR-Egger intercept term. Meanwhile, MR-PRESSO was used to analyze pleiotropy further and pick out outliers in IVs. In addition, to identify potential heterogeneous SNPs, the “leave-one-out” analysis was performed by omitting each instrumental SNP in turn, aiming to verify the reliability and stability of the causal association (Chen et al., 2022).

To further evaluate whether there is a causal relationship between intestinal flora and HSCR, we performed a reverse MR analysis. Bacteria with a significant causal association with positive MR were selected as outcome variables, and HSCR was used as exposure for reverse MR analysis. The analysis methods and SNP selection conditions were consistent with those of positive MR.

False discovery rate (FDR) correction was conducted by applying the q-value procedure, with a FDR q-value of <0.1 (Storey and Tibshirani, 2003). Genera of GM and HSCR were considered to have a suggestive association when *p* < 0.05 but q ≥ 0.1 and a significant association when *p* < q < 0.05.

### 2.5 16S rDNA sequencing

To characterize the bacterial community taxonomically, we performed 16S rDNA amplicon sequencing. First, genomic DNA was extracted from feces using the QIAamp Fast DNA Stool Mini Kit. Illumina-compatible primers (338F, 5′-ACTCCTACGGGAGGCAGCAG-30; 806R, 5′-GGACTACHVGGGTWTCTAAT-30) were then used to amplify the 16S rDNA V3–V4 regions by conventional PCR (Tian et al., 2023). The amplification products were subjected to gel purification prior to quantification by QuantiFluor-ST. After reverse transcription, the cDNA library was processed by fragmentation, end repair, and A-tailing using the TruSeq DNA PCR-free sample preparation kit. Then, the pooled library was run and sequenced on the NovaSeq600. According to QIIM, the effective Tags were obtained after chimera picking and quality filtering. Clustering by Uparse v7.0.1001 was performed to generate a list of open reference operational taxonomic units (OTUs) with an identity of 97%. The taxonomic assignment was subsequently achieved with the SSU rRNA database (Tian et al., 2023).

### 2.6 Targeted mass spectrometry

A targeted mass spectrometry assay was developed to detect the short-chain fatty acid (SCFA) levels in feces. Briefly, acetic, propionic, butyric, isobutyric, valeric, isovaleric, and hexanoic acids were purchased from Sigma Aldrich, as standard solutions with 10 concentration gradients (0.02, 0.1, 0.5, 1, 2, 5, 10, 25, 50, and 100 μg/mL). The sample was mixed with 50 μL of 15% phosphoric acid, 10 μL of 75 μg/mL of isohexanoic acid (internal standard), and 140 μL of diethyl ether, homogenated for 1 min, then centrifuged at 15,000 g at 4°C for 10 min. Next, the supernatant was collected for the test. The Agilent HP-INNOWax column was used for split injection, and the sample volume was 1 μL, the split injection ratio was 10:1, the inlet temperature was 250°C, the ion source temperature was 230°C, the transmission line temperature was 250°C, and the quadrupole temperature was 150°C. The programmed temperature increased from 90°C to 120°C at 10°C/min, next to 150°C at 5°C/min, and finally, to 250°C at 25°C/min for 2 min. The helium carrier gas flow rate was 1.0 mL/min. Mass spectrometry conditions included an electron bombardment ionization (EI) source, SIM scanning mode, and electron energy of 70 eV. The samples and standard solutions of SCFAs were measured by MS. The area under the curve for standard SCFA solution normalized to the internal standard was used as a single point on that SCFA standard curve. A standard curve for each SCFA was established based on the concentration gradient and served to calculate the concentration of this SCFA in the samples. All the samples were measured repeatedly, at least six times (Tian et al., 2023).

### 2.7 Statistics

R software (version 4.3.1) and GraphPad Prism (version 8.0.1) were used to conduct the statistical analysis. We performed MR by the TwoSampleMR (version 0.5.7), MR-PRESSO (version 1.0; Verbanck et al., 2018), q-value (version 2.34.0; Storey and Tibshirani, 2003), and ggplot2 (version 3.3.3) R packages. Normally distributed data were presented as means standard error of the mean (SEM), and non-normally distributed data were presented as medians (interquartile range). Depending on the distribution and variances, a two-tailed Student’s t-test was applied to compare differences between groups. Significance was set at a *p*-value of <0.05. All figure panels represent data independently repeated at least three times, yielding similar results.

## 3 Results

### 3.1 Selection of instrumental variables in forward MR analysis

According to the selection criteria for IVs, 2,699 SNPs (*p* < 1.0 × 10^−5^) were selected for 196 intestinal flora in our MR analysis process. Details of all SNPs are provided in Supplementary Table S1.

### 3.2 MR analysis

**Figure 3:**
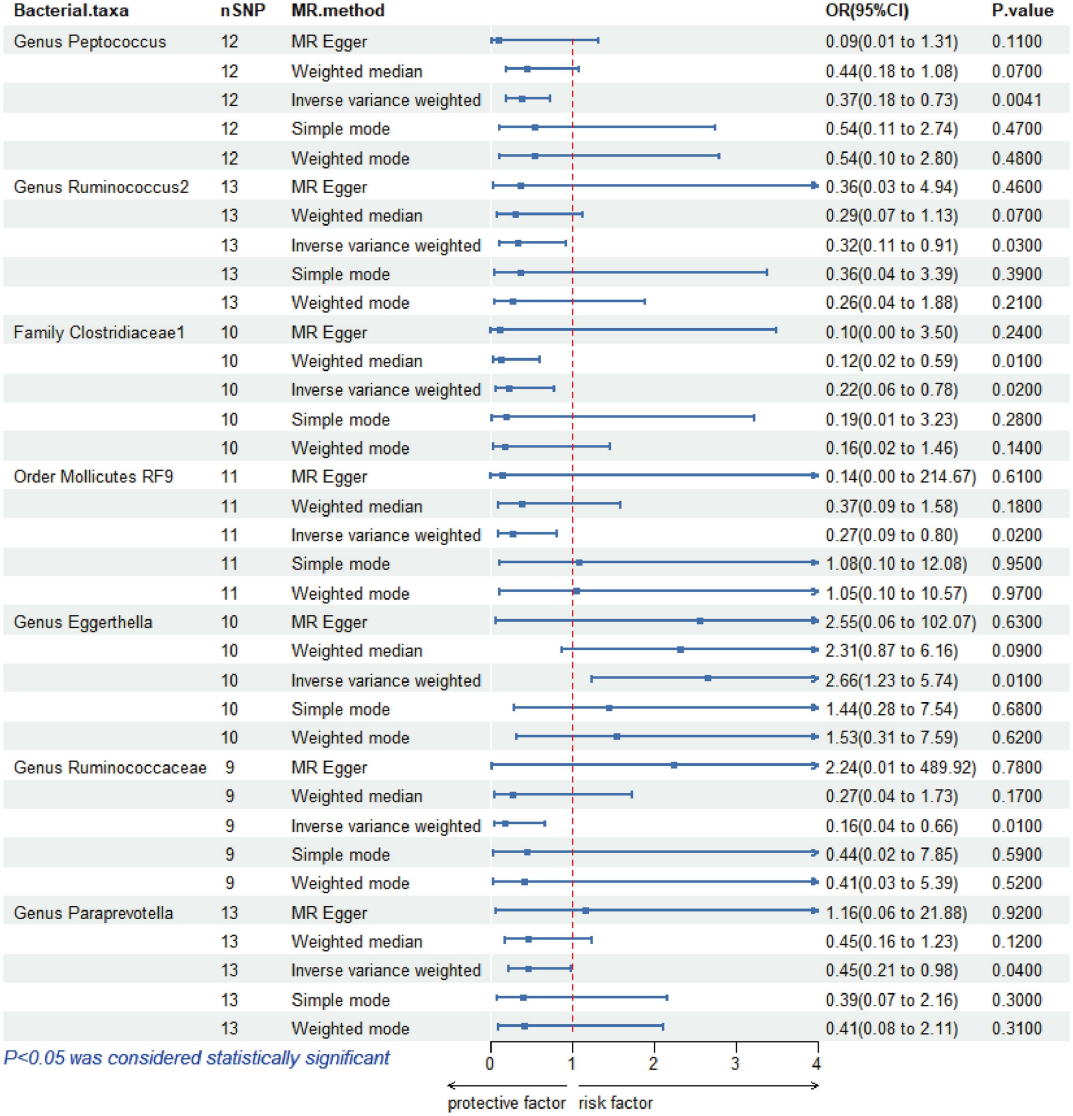
Mendelian randomization analysis of gut microbiota and Hirschsprung disease. OR, odds ratio; 95% CI, 95% confidence interval; MR, Mendelian randomization; SNPs, single-nucleotide polymorphisms.

**Figure 4:**
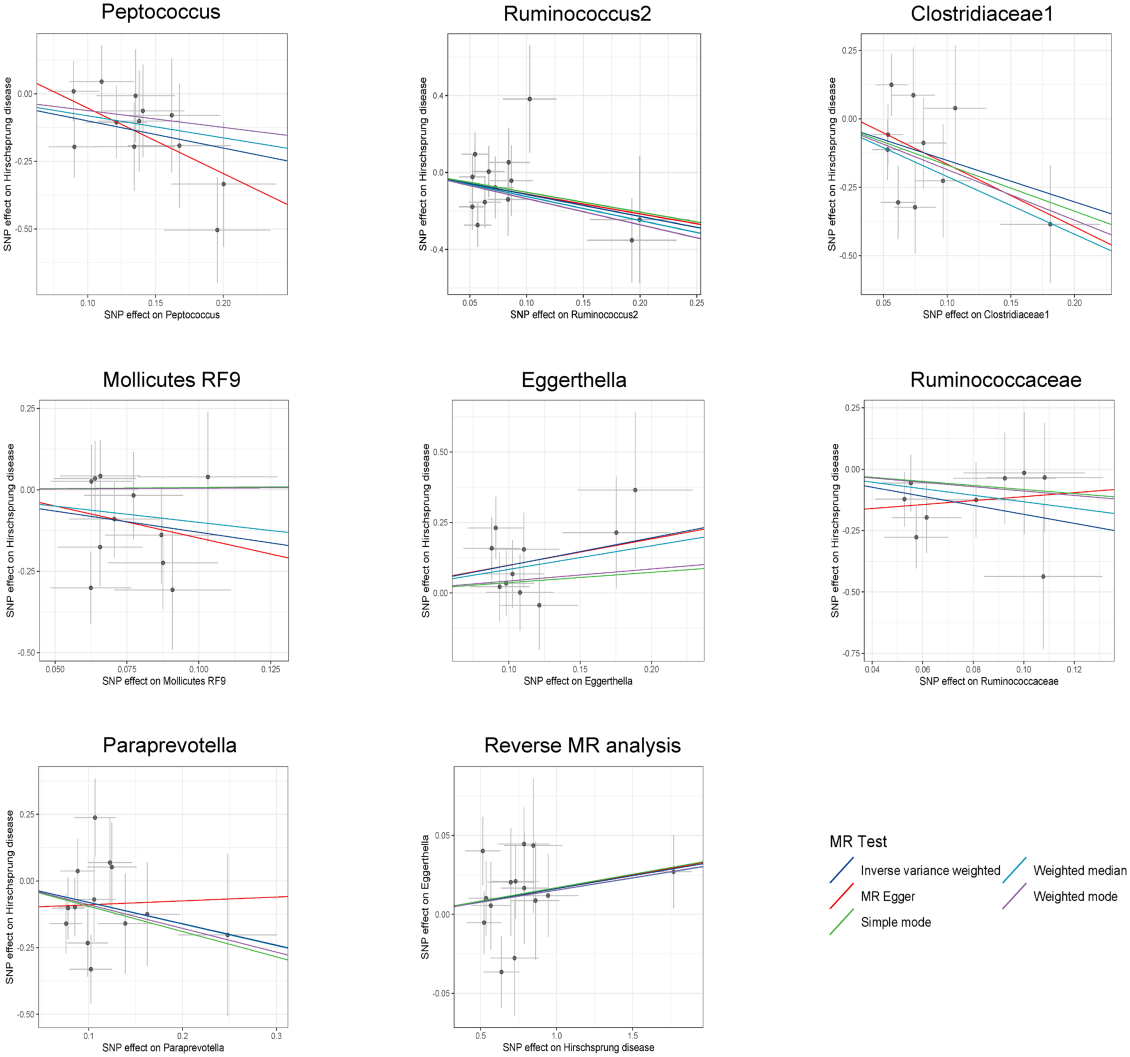
Scatter plots for bidirectional MR analyses of the causal effect of gut microbiota on Hirschsprung disease. SNPs, single-nucleotide polymorphisms; MR, Mendelian randomization.

IVW estimates were employed as the primary MR assay, and seven intestinal flora significantly associated with HSCR were identified. As shown in Figure 3, Eggerthella (OR: 2.66, 95%CI: 1.23–5.74, *p* = 0.01) was associated with an increased risk of HSCR, while Peptococcus (OR: 0.37, 95%CI: 0.18–0.73, *p* = 0.004), Ruminococcus2 (OR: 0.32, 95%CI: 0.11–0.91, *p* = 0.03), Clostridiaceae1 (OR: 0.22, 95%CI: 0.06–0.78, *p* = 0.02), Mollicutes RF9 (OR: 0.27, 95%CI: 0.09–0.8, *p* = 0.02), Ruminococcaceae (OR: 0.16, 95%CI: 0.04–0.66, *p* = 0.01) and Paraprevotella (OR: 0.45, 95%CI: 0.21–0.98, *p* = 0.04) were associated with reduced risks of HSCR. We corrected all *p*-values with FDR, and the Q-values were all greater than 0.05 (Supplementary Table S2). The other four methods are consistent with the direction of the IVW beta value (Figure 4).

### 3.3 Sensitivity analysis

**Figure 5:**
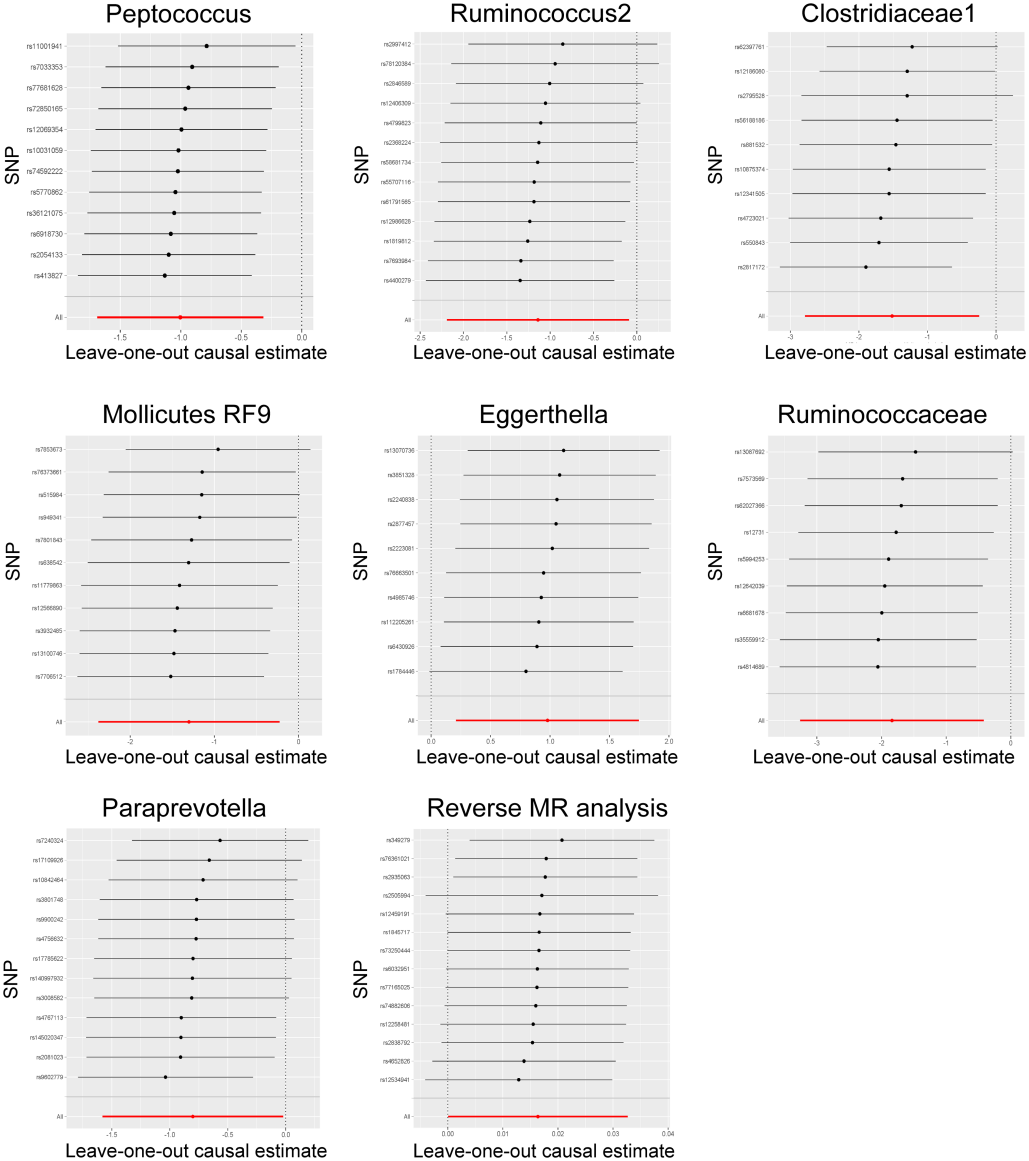
Leave-one-out plots for the bidirectional MR analysis of the causal effect of gut microbiota on Hirschsprung disease. MR, Mendelian randomization; SNPs, single-nucleotide polymorphisms.

Among these seven causal associations, the F-statistics of the IVs ranged from 17.42 to 32.26, eliminating the bias of weak IVs (Supplementary Table S3). As shown in Table 1, Cochran’s Q-test showed no significant heterogeneity for these IVs (*p* > 0.05; Supplementary Table S4), the MR-Egger regression intercept analysis found no horizontal pleiotropy (*p* > 0.05; Supplementary Table S5), and the MR-PRESSO test showed no outliers (global test *p* > 0.05; Supplementary Table S6). Moreover, after correcting the outliers, there is still no pleiotropy, which is supplemented by the MR-Egger regression intercept analysis. Subsequently, by drawing scatter plots (Figure 4) and leave-one-out plots (Figure 5), we did not find significant outliers for all IVs.

**Table 1:** Sensitivity analysis between gut microbiota and Hirschsprung disease.

Bacterial taxa	Heterogeneity test		Pleiotropy test	
	MR-Egger_P	MR-IVW_P	Egger-intercept_P	MR-PRESSO_P
Genus Peptococcus	0.83	0.8	0.31	0.79
Genus Ruminococcus2	0.39	0.48	0.93	0.53
Family Clostridiaceae1	0.27	0.34	0.66	0.40
Order Mollicutes RF9	0.31	0.39	0.86	0.41
Genus Eggerthella	0.76	0.83	0.98	0.86
Genus Ruminococcaceae	0.83	0.8	0.35	0.84
Genus Paraprevotella	0.28	0.31	0.53	0.31

IVW, inverse-variance weighted.

### 3.4 The result of reverse MR analysis

Reverse MR analysis showed that HSCR was associated with an increased abundance of Eggerthella (Supplementary Table S7; Figure 4), while no significant causal association was found with the other six bacteria (Supplementary Table S7). Cochran’s Q-test showed no significant heterogeneity in the IVs of HSCR (*p* > 0.05; Supplementary Table S8). Meanwhile, the analysis results of MR-Egger and MR-PRESSO (*p* > 0.05; Supplementary Table S8) show that horizontal pleiotropy is insignificant.

### 3.5 Detection of microbiota and short-chain fatty acids

**Figure 6:**
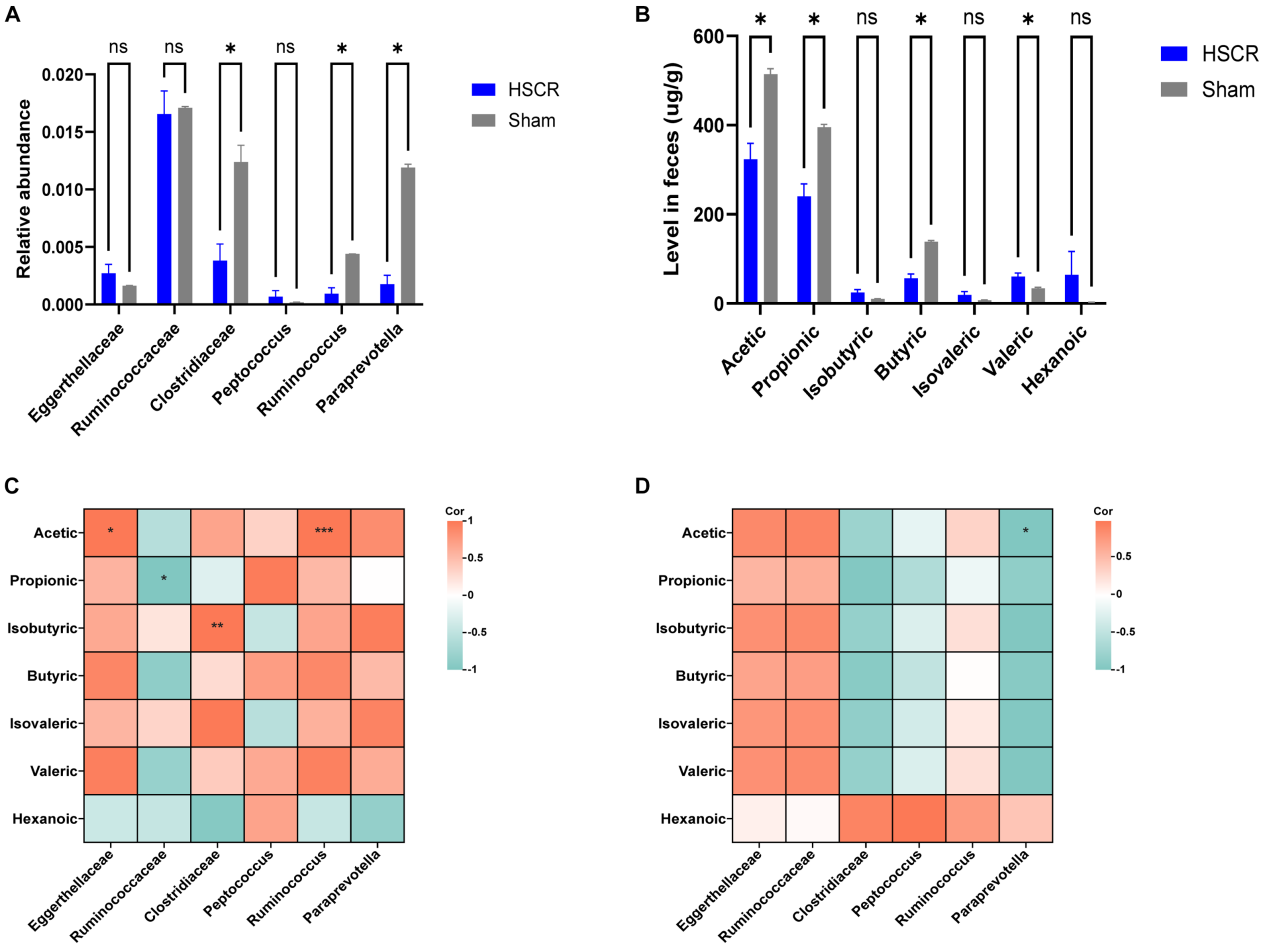
Detection of microbiota and short-chain fatty acids. **(A)** Relative abundance of GM. **(B)** Level of SCFAs in feces. **(C)** Correction analysis of GM and SCFAs in the HSCR group. **(D)** Correction analysis of GM and SCFAs in the control group. HSCR, Hirschsprung disease; GM, gut microbiota; SCFAs, short-chain fatty acids. Cor, correlation coefficient. Data were represented as means ± SEM (*n* = 3 biological replicates per group). ^*^p < 0.05, ^**^p < 0.01, and ^***^p < 0.001.

To investigate the alteration of the significant intestinal flora in HSCR, 16S rDNA sequencing was employed. Compared with the control, the relative abundance of Clostridiaceae (P: 0.002), Ruminococcus (P: 0.0003), and Paraprevotella (P: 0.00003) was significantly decreased (*p* < 0.05), while Eggerthellaceae was increased without statistical significance (*p* < 0.1) in HSCR. There were no significant changes in Ruminococcaceae (P: 0.671) or Peptococcus (P: 0.185; Figure 6A; Supplementary Tables S9, S10).

According to MR and the difference analysis, Clostridiaceae and Ruminococcus, as protective flora, showed a significant decrease in HSCR, and were reported as the producers of SCFAs (Vojinovic et al., 2019; Wu et al., 2023). Thus, we performed metabolically targeted mass spectrometry for SCFAs in fecal samples. Compared with the control, acetic, propionic, and butyric acid levels were lower in the HSCR group, while caproic acid levels were higher (Figure 6B; Supplementary Tables S11, S12). We further analyzed the correlation between significant flora and SCFAs. In the HSCR group, Ruminococcaceae was negatively correlated with acetic acid (R: −0.998, P: 0.041), while Clostridiaceae was positively correlated with isobutyric acid (R: 1.000, P: 0.002), and Ruminococcus was positively correlated with acetic acid (R: 1.000, P: 0.001; Figure 6C; Supplementary Table S13). However, in the control group, only Paraprevotella was negatively associated with acetic acid (R: −1.000, P: 0.012; Figure 6D; Supplementary Table S14).

These results suggested that the protective flora alternation was closely associated with SCFA levels in the intestinal microenvironment of HSCR patients.

## 4 Discussion

HSCR is characterized by abnormal structure and function of the ENS, which is supposed to be dominated by genetic factors (Montalva et al., 2023). HSCR occurs unusually in infants who delay or have no meconium excretion; it is also diagnosed in preschool children (Parathan et al., 2020). There is further development and maturity of ENS after birth (Radenkovic et al., 2018), during which the GM colonizes and changes significantly (Stewart et al., 2018), indicating the microbiota is correlated with HSCR pathogenesis. Studies have shown that the abnormal intestinal microenvironment contributed crucially to the HSCR pathogenesis, especially gut GM, but without details (Obata and Pachnis, 2016; Vincent et al., 2023). In this study, we performed bidirectional two-sample MR to analyze the causal relationship between GM and HSCR, as well as 16S rDNA sequencing and targeted mass spectrometry to characterize the bacterial community taxonomically and metabolically. We found that Eggerthella was associated with an increased risk of HSCR, and HSCR was identified to promote Eggerthella flora colonizing the intestine. Meanwhile, Peptococcus, Ruminococcus, Clostridiaceae, Mollicutes RF9, Ruminococcaceae, and Paraprevotella were identified as protective factors for HSCR, of which the relative abundance of Clostridiaceae, Ruminococcus, and Paraprevotella in HSCR was significantly decreased and was correlated with SCFAs.

The development of the ENS is a gradual process, going through key developmental stages and gradually maturing in the few years after birth (Gershon and Ratcliffe, 2004; Nagy and Goldstein, 2017; Radenkovic et al., 2018; Parathan et al., 2020). Coincidentally, the GM, as a major factor in the intestinal microenvironment, begins to colonize immediately after birth, with significant changes from the neonatal period to early childhood (Backhed et al., 2015; Gritz and Bhandari, 2015; Yassour et al., 2016; Stewart et al., 2018). This may explain why many patients do not have obvious symptoms in early life but occur intestinal obstruction in childhood or even adolescence and are diagnosed with HSCR by internal diameter biopsy (Barnes et al., 1986; Wheatley et al., 1990; Pemmada et al., 2023). Some studies have shown that people with HSCR have different GM than healthy children, and the microbiota diversity is lower (Yan et al., 2014; Li et al., 2016; Neuvonen et al., 2018). For HSCR, some protective flora and its metabolites may promote enteric neural stem cells phenotype, thereby ameliorating the intestinal motivity due to innate genetic mutations (De Vadder et al., 2018; Tian et al., 2023). Conversely, risk factors related to GM may lead to chronic inflammation and affect local intestinal immunity, even intestinal glial cell maturation (Obata and Pachnis, 2016; Behzadi et al., 2024), which all contribute to the HSCR.

SCFAs are one of the main metabolites of GM. In our MR results, some SCFA-producing bacteria, such as Peptococcus (Wang H. et al., 2023), Ruminococcus2 (Wu et al., 2023), Clostridiaceae1 (Vojinovic et al., 2019), and Ruminococcaceae (D'Amato et al., 2020; Zhou et al., 2022) were identified to be protective to HSCR, some of which in HSCR was significantly decreased, and were correlated with SCFAs. Animal studies suggested that SCFAs could independently stimulate the enteric neural stem cell phenotype, thereby improving enteric neural disorders (Fung et al., 2021; Tian et al., 2023; Wang L. et al., 2023). Recent studies revealed that SCFA promoted enterochromaffin cells (ECs) to secrete 5-hydroxytryptamine (5-HT; Atarashi et al., 2013; Furusawa et al., 2013; Reigstad et al., 2015; Yano et al., 2015), which is regulated by the calcitonin selective reuptake transporter (SERT) of epithelial cells and plays crucial roles in enteric neuroregeneration and neuroprotection (Bian et al., 2007). Intestinal SERT is expressed lower in children compared to adults, and therefore 5-HT is more available in the early stages of ENS development and maturity (Bian et al., 2007). Additionally, SCFAs specifically increase the proportion of excitatory cholinergic neurons in the colon and regulate motor function (Soret et al., 2010). Moreover, Ruminococcus2 and Clostridiaceae1 participate in the metabolism of cholic acid (Tanaka et al., 2020; Wang et al., 2020; Molinero et al., 2022), which can activate the G-protein-coupled bile acid receptor (TGR5; Lin et al., 2023). Secondary bile acids have been shown to stimulate secretion of 5-HT, glucagon-like peptide 1 (GLP-1), and calcitonin gene-related peptide (CGRP), all of which significantly regulate the ENS and intestinal motility (Bampton et al., 2002; Kidd et al., 2008; Alemi et al., 2013). These results suggested that the flora (Peptococcus, Ruminococcus, Clostridiaceae, and Ruminococcaceae) with metabolite SCFAs and bile acids contribute to ENS development and functional maturation, which play a potential protective role and thereby reduce the risk of HSCR.

Eggerthella was the only significant factor both in the MR and the reverse MR analyses. In our study, Eggerthella colonization increased the risk of HSCR, and the HSCR patients were more conducive to Eggerthella survival, which was consistent with 16S rDNA sequencing. According to a recent study, Eggerthella could induce intestinal Th17 activation (Alexander et al., 2022), while the pro-inflammatory factor IL-17 significantly inhibited the proliferation and migration and induced apoptosis of enteric neural stem cells (Tian et al., 2023). This implies that Eggerthella may contribute to HSCR by promoting the release of inflammatory factors. In reverse, HSCR patients may promote Eggerthella colonization. This finding provides new clues about the etiological mechanism of HSCR and helps clinical antibiotic selection to prevent and cure Hirschsprung-associated enterocolitis (HAEC).

Moreover, whether and how the microbiome induces HSCR during pregnancy remains unclear. It is reported that the mother’s GM changes significantly during pregnancy (Goltsman et al., 2018) and plays a vital role in fetal health (Di Simone et al., 2020). One animal study confirmed that maternal GM metabolites circulated between the mother and fetus, such as SCFAs, regulate placental growth and blood vessel formation (Pronovost et al., 2023). Furthermore, the inflammatory factors produced by the mother’s gut flora impacted early fetal development (Apostol et al., 2020). Therefore, it is much more valuable to explore how the above six protective GMs reduce the risk of HSCR during pregnancy, just as the Eggerthella increases the risk of HSCR.

This study has some limitations because aggregated data were used rather than raw data, it was not possible to group by fetal, neonatal, and infantile analyses. Therefore, we cannot determine at which growth stage the gut flora most significantly contributes to HSCR, which also prevents us from further exploring the details of the causal association between GM and HSCR. For sensitivity analysis and pleiotropy testing, there seems to be a need for more genetic variants as IVs. Therefore, SNPs in the MR analysis failed to reach the traditional GWAS significance threshold (*p* < 5.0 × 10^−8^). With the gradual improvement of GWAS data, it is suggested that future MR studies can be stratified based on pre-birth and post-birth studies to obtain more precise and complete results. Furthermore, MR has the advantage of evaluating the direct effect of exposure factors on the outcome, while intermediate effects or effects via other pathways may be ignored (Sekula et al., 2016). All the above may limit the full understanding of the causal chain, which needs to be investigated further.

## 5 Conclusion

In conclusion, our bidirectional two-sample MR study demonstrates a causal association between GM and HSCR. Specifically, Peptococcus, Ruminococcus, Clostridiaceae, Mollicutes RF9, Ruminococcaceae, and Paraprevotella are protective factors against HSCR, while Eggerthella may increase the risk for HSCR. Moreover, reverse MR also confirmed that HSCR was a risk factor for Eggerthella. 16S rDNA sequencing and targeted mass spectrometry presented significant alteration of Clostridiaceae, Ruminococcus, and Paraprevotella, as well as correlation with SCFAs, which underline the importance of further study and provide new insights into the pathogenesis and treatment. However, the details need further study.

## Data availability statement

The original contributions presented in the study are included in the article/Supplementary material, further inquiries can be directed to the corresponding authors.

## Author contributions

WL: Data curation, Methodology, Validation, Visualization, Writing – original draft. HYa: Software, Supervision, Visualization, Writing – review & editing. WJ: Formal analysis, Methodology, Resources, Writing – review & editing. JH: Conceptualization, Investigation, Validation, Writing – review & editing. ZF: Methodology, Software, Writing – review & editing. WX: Data curation, Methodology, Validation, Writing – review & editing. HYu: Funding acquisition, Software, Writing – review & editing. WY: Data curation, Methodology, Writing – review & editing. WP: Supervision, Validation, Writing – review & editing. BZ: Investigation, Writing – review & editing. YL: Investigation, Writing – review & editing. XC: Investigation, Writing – review & editing. YG: Funding acquisition, Methodology, Supervision, Writing – review & editing. DT: Funding acquisition, Investigation, Supervision, Writing – review & editing.
